# lncRNA LIFR-AS1 suppresses invasion and metastasis of non-small cell lung cancer via the miR-942-5p/ZNF471 axis

**DOI:** 10.1186/s12935-020-01228-5

**Published:** 2020-05-24

**Authors:** Qun Wang, Jing Wu, Hui Huang, Yan Jiang, Ying Huang, Hongyan Fang, Gang Zheng, Xiaochun Zhou, Yujuan Wu, Changjiang Lei, Desheng Hu

**Affiliations:** 1 Department of Radiotherapy, Hubei Cancer Hospital, Tongji Medical College, Huazhong University of Science and Technology, 116 Zhuodaoquan South Road, Wuhan, 430070 China; 2 Department of Oncology, The Fifth Hospital of Wuhan, Wuhan, China; 3 WuHan University, Wuhan, China; 4 Department of Pneumology, The Fifth Hospital of Wuhan, Wuhan, China; 5 Department of General Surgery, The Fifth Hospital of Wuhan, Wuhan, China

**Keywords:** Lung cancer, lncRNA, Metastasis, miR-942-5p, Target gene

## Abstract

**Background:**

MicroRNA 942-5p (miR-942-5p) has been reported to promote migration and invasion in non-small cell lung cancer (NSCLC), but the underlying mechanism is not completely understood. The interplay between long non-coding RNAs (lncRNAs) and miRNAs plays a crucial role in tumor progression.

**Methods:**

In the present study, we performed bioinformatic and biochemical analyses to identify miR-942-5p-interacting lncRNAs. The function and clinical significance of the candidate lncRNA(s) in NSCLC were determined.

**Results:**

We identified LIFR-AS1 as a pivotal miR-942-5p-interacting lncRNA. Overexpression of miR-942-5p caused a reduction of LIFR-AS1 in NSCLC cells. LIFR-AS1 showed the ability to sponge miR-942-5p, leading to derepression of ZNF471. Functionally, LIFR-AS1 overexpression inhibited NSCLC cell migration and invasion, whereas LIFR-AS1 silencing yielded an opposite effect. In vivo studies confirmed that LIFR-AS1 overexpression suppressed lung metastasis of NSCLC cells. Rescue experiments demonstrated that enforced expression of miR-942-5p or depletion of ZNF471 restored the migration and invasion capacity of LIFR-AS1-overexpressing cells. Moreover, overexpression of ZNF471 restrained NSCLC cell invasion. Clinically, LIFR-AS1 downregulation was significantly correlated with TNM stage, lymph node metastasis, and reduced overall survival in NSCLC patients.

**Conclusions:**

we provide first evidence for the involvement of the LIFR-AS1/miR-942-5p/ZNF471 axis in NSCLC invasion and metastasis. LIFR-AS1 may represent a novel target for the treatment of NSCLC.

## Background

Non-small cell lung cancer (NSCLC), accounting for more than 85% of all lung cancers, is one of the leading causes of cancer-related mortality [1]. The 5-year overall survival rate for patients with NSCLC is approximately 15% [2]. The poor prognosis of NSCLC is mainly attributed to cancer cell invasion and metastasis [3]. Hence, exploring the molecular mechanism underlying NSCLC metastasis is of significance in treating this malignancy.

Zinc-finger protein 471 (ZNF471), belonging to the large family of zinc-finger proteins, is frequently downregulated in tumors due to promoter hypermethylation [4, 5]. Bhat et al. [4] demonstrated that downregulation of ZNF471 is significantly associated with reduced survival in patients with head and neck squamous cell carcinoma. ZNF471 has exhibited tumor-suppressive activities in gastric cancer [5] and esophageal cancer [6]. These studies indicate ZNF471 as a tumor suppressor.

Long non-coding RNAs (lncRNAs) represent a large class of non-coding RNAs of longer than 200 nucleotides [7]. In recent years, lncRNAs have attracted attention as important regulators of biological processes such as cell proliferation, differentiation, inflammation, infection, and tumor progression [7–9]. Most interestingly, lncRNAs can coordinate NSCLC growth and metastasis [8, 9]. For example, the lncRNA GIAT4RA has been reported to inhibit NSCLC cell growth, colony formation, migration, and invasion [8]. *LIFR*-*AS1* is located on human chromosome 5p13.1. A previous study reported that LIFR-AS1 inhibits the proliferation and survival of colorectal cancer cells [10]. Another study demonstrated that LIFR-AS1 has the ability to control breast cancer cell proliferation and migration [11]. However, the role of LIFR-AS1 in NSCLC remains unclear.

It has been suggested that lncRNAs can exert their biological effects by sponging microRNAs (miRNAs) to regulate target gene expression [9]. miRNAs are small, endogenous non-coding RNAs that are capable of repressing gene expression by binding to the 3′-untranslated region (3′-UTR) of target mRNAs [10]. Compelling evidence indicates that lncRNAs can work as competitive endogenous RNA (ceRNA) for miRNAs [9, 11, 12]. For example, the lncRNA MALAT1 antagonizes the activity of miR-199a to promote ZHX1 expression, leading to increased glioblastoma cell proliferation and survival [12].

miR-942-5p plays an oncogenic role in multiple cancer types including esophageal squamous cell cancer [13], colorectal cancer [14], hepatocellular carcinoma [15], and breast cancer [16]. Yang et al. [17] confirm the tumor-promoting activity of miR-942-5p in NSCLC. The lncRNAs ADAMTS9-AS2 [18] and LINC00675 [14] have been reported to sponge miR-942-5p in mesenchymal stem cells and colorectal cancer cells, respectively. However, overexpression of either of the 2 lncRNAs did not affect the expression of miR-942-5p in NSCLC cells (data not shown). Thereby, in the present study, we aim to identify novel miR-942-5p-interacting lncRNAs and explore their function in NSCLC.

## Methods and materials

### Cell culture

Human NSCLC cell lines A549, H1299, PC-9, and H1975 and human bronchial epithelial cell line BEAS-2B were purchased from the Cell Bank of Shanghai Institute of Cell Biology, Chinese Academy of Sciences (Shanghai, China). NSCLC cells were cultured in Dulbecco’s modified Eagle Medium (DMEM, Invitrogen, Carlsbad, CA, USA) with 10% fetal bovine serum (FBS; Invitrogen). BEAS-2B cells were grown in growth factor-supplemented medium (BEGM; Lonza, Walkersville, MD, USA). All the cell lines were maintained in a humidified atmosphere of 5% CO_2_ at 37 °C.

### Oligonucleotides, plasmids, and transfections

MiR-942-5p mimic and negative control mimic were purchased from Sigma-Aldrich (St. Louis, MO, USA). The sequences of LIFR-AS1 (NR_103553.1) and ZNF471 (NM_020813.4) were inserted to pcDNA3.1(+) expression vector. Short hairpin RNAs (shRNAs) targeting LIFR-AS1 and ZNF471 were synthesized by Shanghai Sangon Company (Shanghai, China) and cloned to the pLKO.1 vector. The wild-type LIFR-AS1 and ZNF471 3′-UTR luciferase reporters were constructed in the pmirGLO plasmid (Promega, Madison, WI, USA). The QuikChange Site-Directed Mutagenesis Kit (Stratagene, La Jolla, CA, USA) was utilized to generate a mutated LIFR-AS1 or ZNF471 3′-UTR with disruption of the putative miR-942-5p binding site. All the constructs were validated by sequencing. They were transfected to NSCLC cell lines using Lipofectamine 3000 transfection reagent (Invitrogen) according to the manufacturer’ s instruction.

### Quantitative real-time PCR (qRT-PCR)

Total RNA was extracted from tissues and cells using TRIzol Reagent (Invitrogen). Reverse transcription was carried out using the QuantiTect Reverse Transcription Kit (Qiagen, Hilden, Germany). The resultant cDNA was amplified using a SYBR Green PCR Kit (Qiagen). The PCR primers used are as follows: LIFR-AS1 forward 5′-GCAAATACTGTGTATTAGTCC-3′ and LIFR-AS1 reverse 5′-CCGCTTCCTTGTGAAGAAGGT-3′; ZNF471 forward 5′-CACAGCTGGCTACTCATCAGA-3′ and ZNF471 reverse 5′-GCTGAAGGCTTTCCCGCATTC-3′; RND3 forward 5′-CTATGACCAGGGGGCAAATA-3′ and RND3 reverse 5′-TCTTCGCTTTGTCCTTTCGT-3′; CCBE1 forward 5′-AGGCGACACTCCACAGT-3′ and CCBE1 reverse 5′-GATTAGTGGTCGCTATATT-3′; KDM5A forward 5′-GATGACAGCATGGAAGAGAAAC-3′ and KDM5A reverse 5′-GCCAGTTTATTCAGCTCCTTTG-3′; GAPDH forward 5′-ACCACAGTCCATGCCATCAC-3′ and GAPDH reverse 5′-TCCACCACCCTGTTGCTGTA-3′. For detection of miR-942-5p, cDNA was synthesized by the TaqMan MicroRNA Reverse Transcription Kit (Applied Biosystems, Foster City, CA, USA)according to the manufacturer’s instruction. RNA expression was quantified using the mirVana qRT-PCR microRNA Detection Kit (Applied Biosystems). U6 was used as the normalization control. The relative expression levels of the target genes were determined by the 2^−ΔΔCt^ method [19].

### Luciferase reporter assay

Luciferase reporter assay was performed as described previously [20]. The LIFR-AS1 and ZNF471 3′-UTR luciferase vectors were co-transfected with miR-942-5p mimic or negative control mimic to NSCLC cells using Lipofectamine 3000. The cells were collected 48 h after transfection, and the luciferase activities were measured using the Dual-Luciferase Reporter Assay System (Promega). The relative luciferase activity was presented after normalization to the activity of *Renilla* luciferase.

### RNA immunoprecipitation (RIP) assay

RIP assay was performed as described previously [21]. In brief, A549 and H1299 cells were lysed using RIP immunoprecipitation buffer containing protease/RNase inhibitors (Roche, Madison, WI, USA). In some settings, A549 cells were transfected with miR-942-5p mimic or control mimic together with wild-type or mutated ZNF471. The lysates were then subjected to immunoprecipitation using anti-Ago2 monoclonal antibody or mouse IgG control (Sigma-Aldrich) conjugated to magnetic beads. After incubation at 4 °C overnight, the immunoprecipitated RNA was extracted with Trizol Reagent from the beads and quantitated by qRT-PCR analysis.

### Western blot analysis

Cells were lysed using radioimmunoprecipitation assay buffer with a protease inhibitor cocktail (Roche). Protein concentrations were measured using the Pierce BCA Protein Assay Kit (Thermo Fisher Scientific, Waltham, MA, USA). Protein samples were resolved by SDS–polyacrylamide gel electrophoresis. Rabbit anti-ZNF471/ERP1 (ab204974) and anti-β-actin (ab8227) antibodies (Abcam, Cambridge, MA, USA) were used as the primary antibodies. Protein band was visualized by ECL chemiluminescent reagent (Millipore, Billerica, MA, USA).

### Wound-healing assay

Cell migration was determined using wound-healing assay, as described previously [22]. In brief, transfected cells were plated into 6-well plates and grown to confluence. The cell monolayers were wounded with a pipette tip, and cell debris was rinsed off. 10 μg/ml mitomycin C (Sigma-Aldrich) was added to inhibit cell proliferation. Representative images were captured at 0 and 48 h after injury. The percentage of wound healing was quantified. All experiments were repeated three times.

### Transwell invasion assay

Transwell invasion assay was conducted as described previously [23]. In brief, 5 × 10^4^ cells in serum-free medium were plated into the upper chamber pre-coated with Matrigel (BD Biosciences, San Jose, CA, USA). The lower chambers were added with DMEM supplemented with 10% FBS. After incubation for 48 h at 37 °C, the cells that had invaded were fixed with 4% paraformaldehyde and stained with 0.5% crystal violet. Cells were counted in 5 representative fields per insert under a microscope.

### Animal experiments

For in vivo lung metastasis assay, luciferase-labeled NSCLC cells were intravenously injected into nude mice as described previously [24]. In brief, LIFR-AS1-overexpressing or empty vector-transfected PC-9 cells were stably transfected with a luciferase expression construct. The transfected cells (2 × 10^6^) were injected through the tail vein. Luciferase activity was monitored using the IVIS Spectrum imaging system (Perkin-Elmer Life Sciences, Waltham, MA, USA). Bioluminescence images were acquired 6 weeks after cell injection. The mice were then euthanized, and the lung tissues were harvested and subjected to hematoxylin and eosin (H&E) staining.

### Tissue specimens

We collected 73 pairs of NSCLC and corresponding normal lung tissues from patients with NSCLC who underwent surgical resection between July 2017 and July 2018. None received any anticancer treatment before surgery. Clinicopathological information is shown in Table 1.

**Table 1:** Correlation between LIFR-AS1 expression and clinicopathological features of NSCLC patients (n = 73)

Variable	n	LIFR-AS1		*P*
		Low expression (n = 43)	High expression (n = 30)	
Age				0.8340
< 65	33	19	14	
≥ 65	40	24	16	
Sex				0.4716
Male	52	32	10	
Female	21	11	10	
TNM stage				0.0321
I–II	48	24	24	
III–IV	25	19	6	
Lymph node metastasis				0.0047
Negative	47	22	25	
Positive	26	21	5	

### Statistical analysis

All values are reported as mean ± standard deviation and analyzed by the Student’s *t* test or one-way analysis of variance followed by the post hoc Tukey’s test. The correlation of LIFR-AS1 expression with clinicopathological characteristics was calculated with the Chi square test. *P* < 0.05 was considered statistically significant.

## Results

### LIFR-AS1 is reduced by miR-942-5p in NSCLC cells

**Fig. 1:**
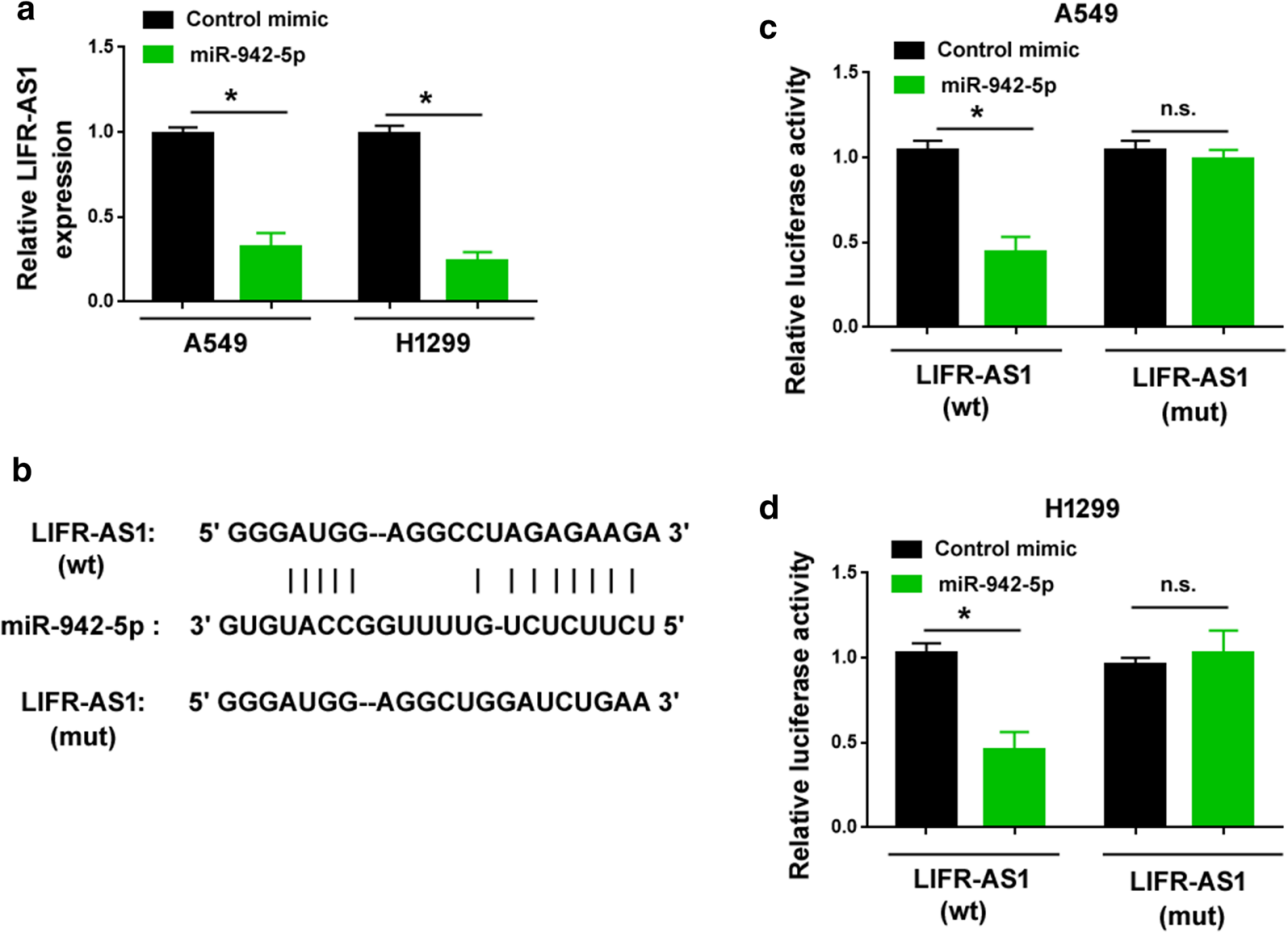
LIFR-AS1 is reduced by miR-942-5p in NSCLC cells. **a** Downregulation of LIFR-AS1 by ectopic expression of miR-942-5p. **b** Prediction of a putative binding site for miR-942-5p in wild-type (wt) LIFR-AS1. Mutated (mut) LIFR-AS1 showed the disruption of miR-942-5p binding site. **c**, **d** Luciferase reporter assay performed in both A549 and H1299 cells revealed that miR-942-5p overexpression repressed the luciferase reporter harboring the wild-type LIFR-AS1 fragment. ^*^P < 0.05; n.s. indicates no significance

To identify miR-942-5p-interacting lncRNAs in NSCLC, we examined the effect of miR-942-5p overexpression on the expression of 21 novel candidates (Additional file 1: Table S1) in A549 cells. These candidate lncRNAs were predicted using the Encyclopedia of RNA Interactomes (ENCORI) program (http://starbase.sysu.edu.cn/index.php). As shown in Fig. 1a, only LIFR-AS1 was downregulated by ectopic expression of miR-942-5p. In contrast, the other 20 lncRNAs tested remained unchanged (data not shown). We also validated the miR-942-5p-induced downregulation of LIFR-AS1 in another NSCLC cell line, H1299 (Fig. 1a). These findings indicate a negative regulation of LIFR-AS1 by miR-942-5p. However, LIFR-AS1 overexpression did not alter the expression of miR-942-5p (Additional file 1: Figure S1).

Bioinformatic analysis predicted a putative binding site for miR-942-5p in LIFR-AS1 (Fig. 1b). To validate the direct interaction between LIFR-AS1 and miR-942-5p, we transfected the LIFR-AS1 luciferase reporters together with miR-942-5p mimic into A549 and H1299 cells. The results showed that miR-942-5p overexpression repressed the luciferase reporter harboring the wild-type LIFR-AS1 fragment, while had no effect on the mutated LIFR-AS1 luciferase reporter (Fig. 1c, d). Altogether, these data confirm that miR-942-5p can target LIFR-AS1 in NSCLC cells.

### LIFR-AS1 sponges miR-942-5p to upregulate ZNF471

**Fig. 2:**
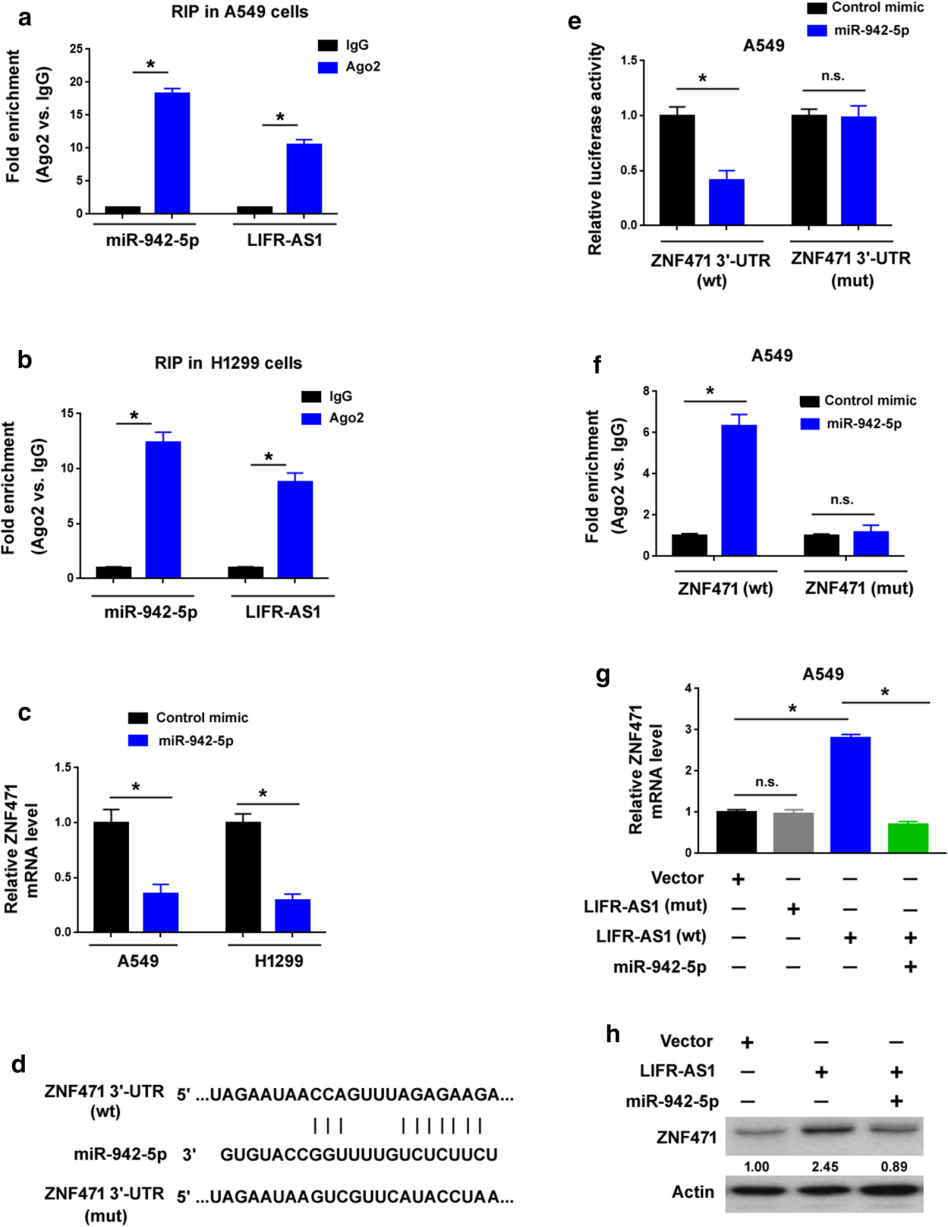
LIFR-AS1 sponges miR-942-5p to upregulate ZNF471. **a**, **b** RIP assays performed with anti-Ago2 antibody showed that miR-942-5p and LIFR-AS1 were detected in Ago2 immunoprecipitates from both A549 and H1299 cells. **c** Overexpression of miR-942-5p decreased the mRNA level of ZNF471 in both A549 and H1299 cells. **d** Bioinformatic analysis predicted that the wild-type (wt) 3′-UTR of ZNF471 harbored a potential miR-942-5p binding site. A mutated (mut) ZNF471 3′-UTR was constructed by disruption of the miR-942-5p binding site. **e** Luciferase reporter assays showed that overexpression of miR-942-5p decreased the luciferase activity of the reporter with the wild-type but not mutated ZNF471 3′-UTR. **f** Ago2 RIP assays were performed in A549 cells transfected with miR-942-5p mimic together with wild-type or mutated ZNF471. Results are expressed as fold enrichment relative to control mimic. **g** Wild-type but not mutated LIFR-AS1 promoted the mRNA expression of ZNF471, which was reversed by miR-942-5p overexpression. **h** Western blot analysis of ZNF471 protein levels in A549 cells transfected with indicated constructs. ^*^P < 0.05; n.s. indicates no significance

It is well known that miRNAs regulate gene expression through the miRNA-induced silencing complex (miRISC), which comprises the effector molecules, argonaute (Ago) proteins [25]. To confirm the binding of miR-942-5p to LIFR-AS1, we performed RIP assays in A549 and H1299 cells using anti-Ago2 antibody. Of note, miR-942-5p and LIFR-AS1 were detected in Ago2 immunoprecipitates, as determined by qRT-PCR analysis (Fig. 2a, b). This result raises the possibility that LIFR-AS1 might act as a sponge of miR-942-5p, thereby regulating target gene expression. A previous study has suggested BARX2 as a direct target of miR-942-5p [17]. However, we did not observe the regulation of BARX2 by LIFR-AS1 in NSCLC cells (Additional file 1: Figure S2). Therefore, we investigated the impact of LIFR-AS1 on other miR-942-5p target genes. Based on the TargetScan program (http://www.targetscan.org/vert_71/), we predicted a large number of target genes for miR-942-5p. Among them, we focused on the genes that were positively correlated with LIFR-AS1 based on The Cancer Genome Atlas (TCGA) data. We then selected the top 25 candidates for further validation. Intriguingly, overexpression of miR-942-5p led to a significant decline in the mRNA level of ZNF471 in both A549 and H1299 cells (Fig. 2c), but had no impact on the other 24 mRNAs including RND3, KDM5A, and CCBE1 (Additional file 1: Figure S3).

To verify the binding between ZNF471 and miR-942-5p, we performed luciferase reporter assays using the luciferase reporter carrying the 3′-UTR of ZNF471 (Fig. 2d, e). Overexpression of miR-942-5p decreased the luciferase activity of the reporter with the wild-type ZNF471 3′-UTR, but not that with a mutated ZNF471 3′-UTR (Fig. 2e). To validate the binding of miR-942-5p to ZNF471 mRNA, we performed Ago2 RIP assays in A549 cells transfected with miR-942-5p mimic or control mimic together with wild-type or mutated ZNF471. The enrichment of wild-type but not mutated ZNF471 in Ago2 immunoprecipitates was observed when miR-942-5p was overexpressed in A549 cells (Fig. 2f). In addition, qRT-PCR analysis indicated that ZNF471 mRNA expression was significantly upregulated in LIFR-AS1-overexpressing NSCLC cells (Fig. 2g). Such upregulation was reversed by overexpression of miR-942-5p (Fig. 2g). However, a mutant LIFR-AS1 with disrupted miR-942-5p binding did not affect the abundance of ZNF471 mRNA. LIFR-AS1-mediated induction of ZNF471 expression was also validated at the protein level (Fig. 2h). These data collectively suggest that LIFR-AS1 can competitively bind with miR-942-5p to derepress ZNF471 in NSCLC cells.

### LIFR-AS1 suppresses NSCLC cell invasion and metastasis

**Fig. 3:**
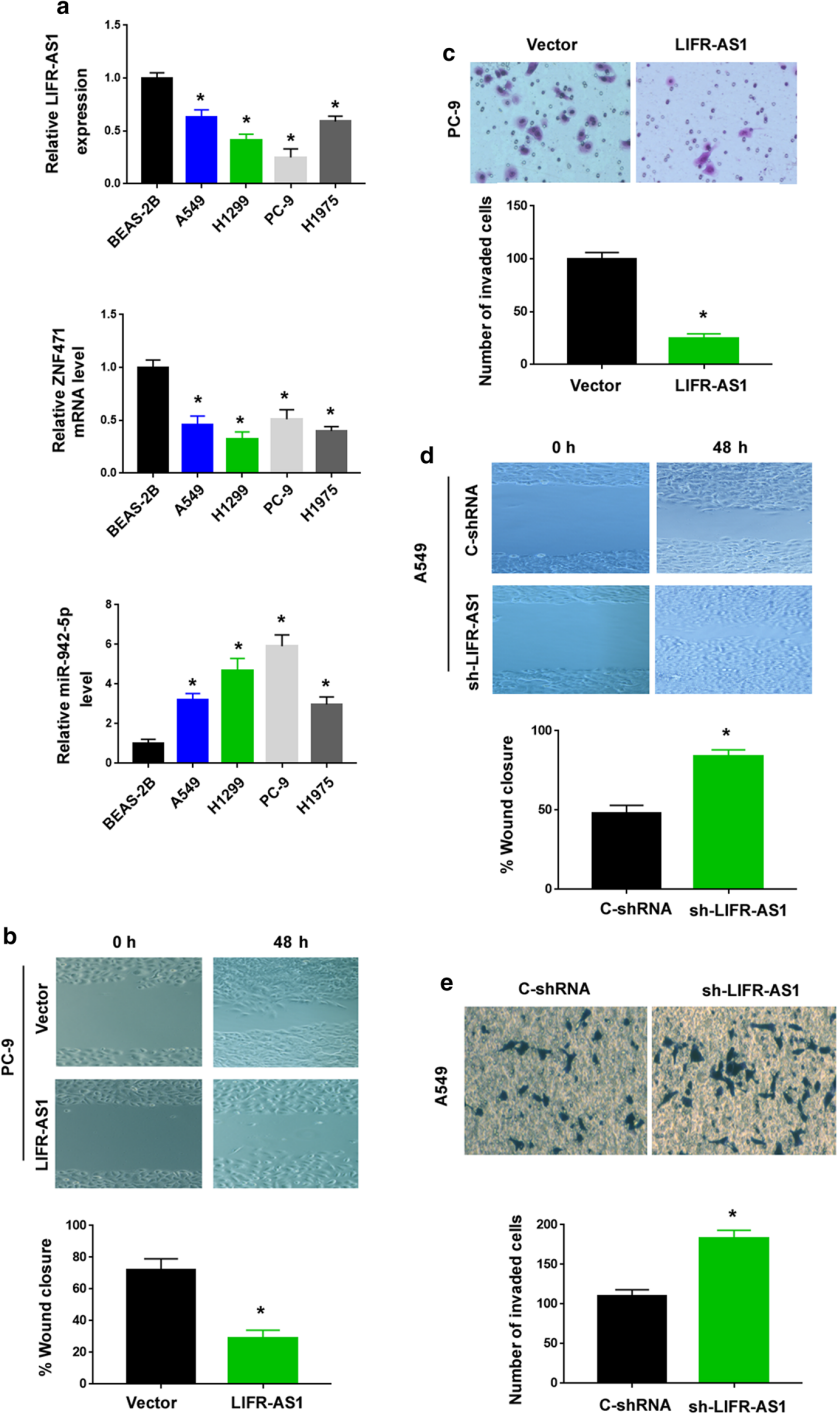
LIFR-AS1 suppresses NSCLC cell migration and invasion. **a** Analysis of LIFR-AS1, ZNF471, and miR-942-5p levels in NSCLC cell lines and BEAS-2B cells. ^*^P < 0.05 vs. BEAS-2B cells. **b** Wound-healing assays showed that overexpression of LIFR-AS1 suppressed the migration of PC-9 cells. **c** Transwell invasion assays revealed that overexpression of LIFR-AS1 inhibited the invasion of PC-9 cells. **d** Knockdown of LIFR-AS1 promoted the migration of A549 cells. **e** Silencing of LIFR-AS1 enhanced the invasion of A549 cells. ^*^P < 0.05

To dissect the role of LIFR-AS1 in NSCLC progression, gain- and loss-of-function experiments were carried out. qRT-PCR analysis revealed that all the 4 NSCLC cell lines tested had significantly lower levels of LIFR-AS1 and ZNF471 than BEAS-2B cells (Fig. 3a). Conversely, the levels of miR-942-5p were significantly increased in NSCLC cell lines compared to BEAS-2B cells. Ectopic expression of LIFR-AS1 inhibited cell migration and invasion in PC-9 cells (Fig. 3b, c), where there was a low level of endogenous LIFR-AS1. Conversely, silencing of LIFR-AS1 caused a promotion of cell migration and invasion in A549 cells (Fig. 3d, e). We also explored the influence of LIFR-AS1 on NSCLC cell proliferation. As determined by MTT assays, overexpression or knockdown of LIFR-AS1 did not alter the proliferation capacity of NSCLC cells (Additional file 1: Figure S4).

**Fig. 4:**
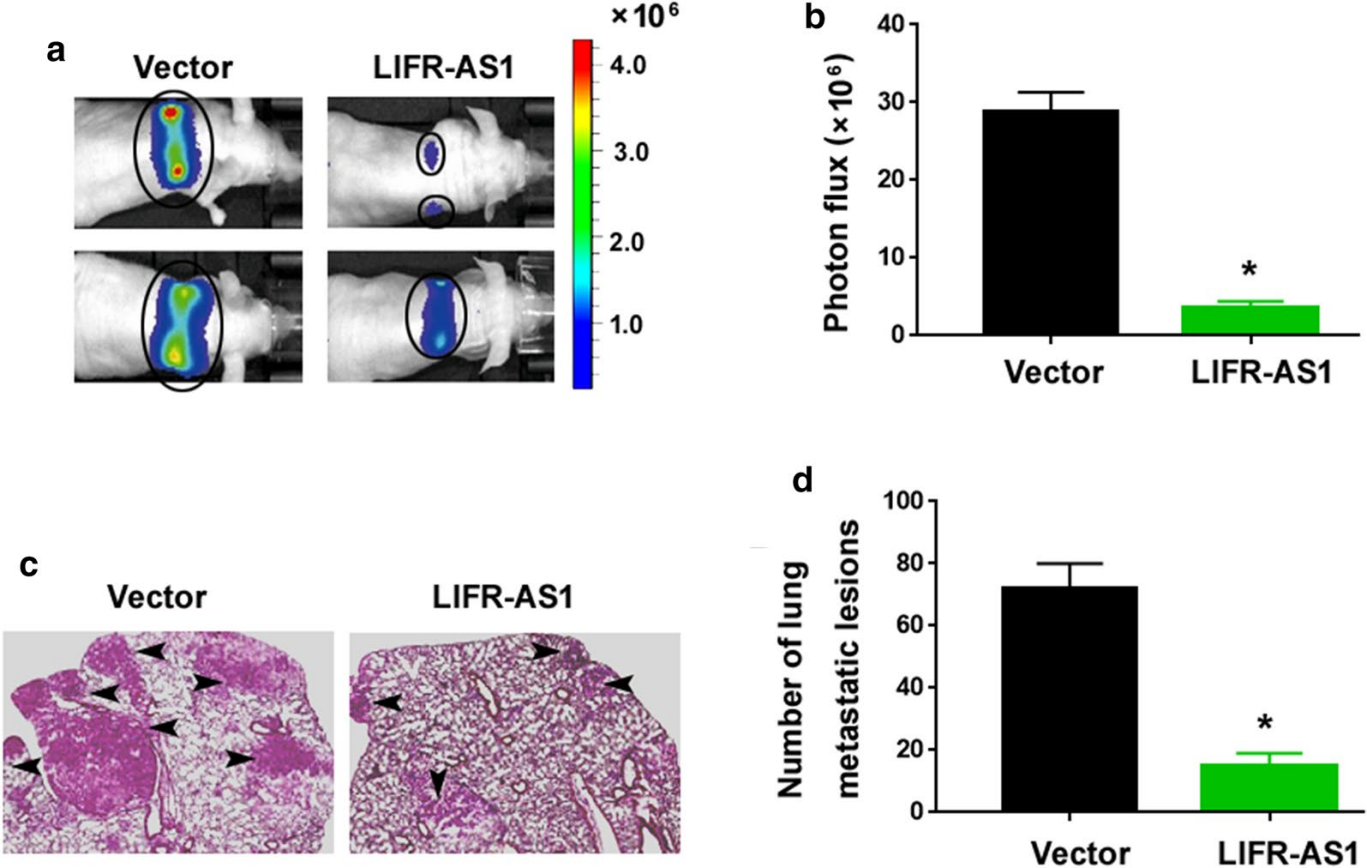
LIFR-AS1 suppresses NSCLC metastasis in vivo. **a**, **b** LIFR-AS1-overexpressing and control PC-9 cells were injected to nude mice through the tail vein. Bioluminescence images were captured 6 weeks after cell injection. **c**, **d** Histological examination showed LIFR-AS1 overexpression resulted in reduced numbers of metastatic lesions in the lung. Scale bar = 50 μm. ^*^P < 0.05 vs. the vector group

To evaluate the effect of LIFR-AS1 on tumor metastasis in vivo, we injected the indicated PC-9 cells into nude mice through the tail vein. Analysis of metastatic activity by bioluminescence imaging revealed that LIFR-AS1-overexpressing PC-9 cells showed decreased lung metastatic activity compared to control cells (Fig. 4a, b). Histological examination further demonstrated that LIFR-AS1 overexpression resulted in reduced numbers of metastatic lesions in the lung (Fig. 4c, d). Taken together, these data indicate that LIFR-AS1 suppresses NSCLC metastasis.

### LIFR-AS1 exerts anti-invasive activity in NSCLC by sponging miR-942-5p and inducing ZNF471

**Fig. 5:**
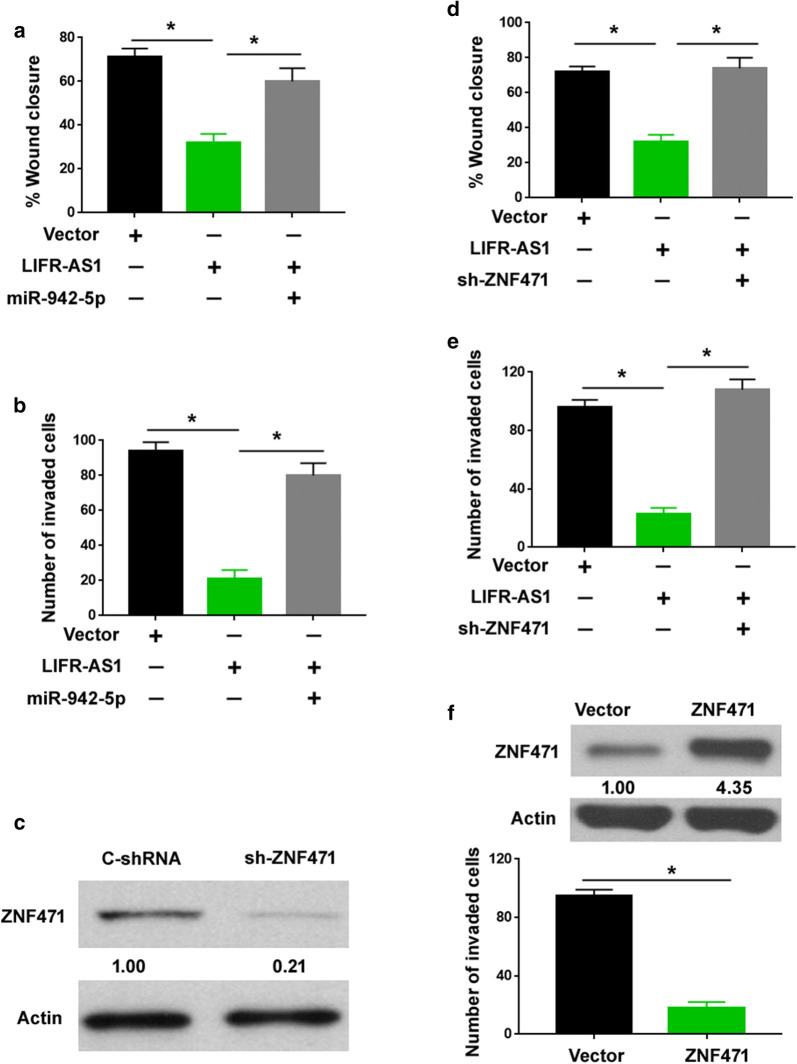
LIFR-AS1 exerts anti-invasive activity in NSCLC by sponging miR-942-5p and inducing ZNF471. **a**, **d** PC-9 cells were transfected with indicated constructs and subjected to wound-healing assays. **b**, **e** PC-9 cells were transfected with indicated constructs and subjected to Transwell invasion assays. **c** Western blot analysis of ZNF471 protein levels in PC-9 cells transfected with control shRNA (C-shRNA) or ZNF471-targeting shRNA (sh-ZNF471). **f** Overexpression of ZNF471 inhibited the invasion of PC-9 cells. ^*^P < 0.05

Next, we asked whether the miR-942-5p/ZNF471 axis was involved in LIFR-AS1-mediated suppression of NSCLC cell invasion. To this end, we performed rescue experiments by overexpressing miR-942-5p or knocking down ZNF471 in LIFR-AS1-overexpressed PC-9 cells. We found that enforced expression of miR-942-5p significantly restored the migration and invasion capacity of LIFR-AS1-overexpressing cells (Fig. 5a, b). Similarly, depletion of ZNF471 (Fig. 5c) reversed the inhibitory effect of LIFR-AS1 on the migration (Fig. 5d) and invasion (Fig. 5e) of PC-9 cells. In addition, overexpression of ZNF471 inhibited the invasion of PC-9 cells (Fig. 5f). Collectively, these observations suggest that ZNF471 acts as a downstream effector of LIFR-AS1 in restraining NSCLC cell invasion.

### Clinical significance of LIFR-AS1 in NSCLC

**Fig. 6:**
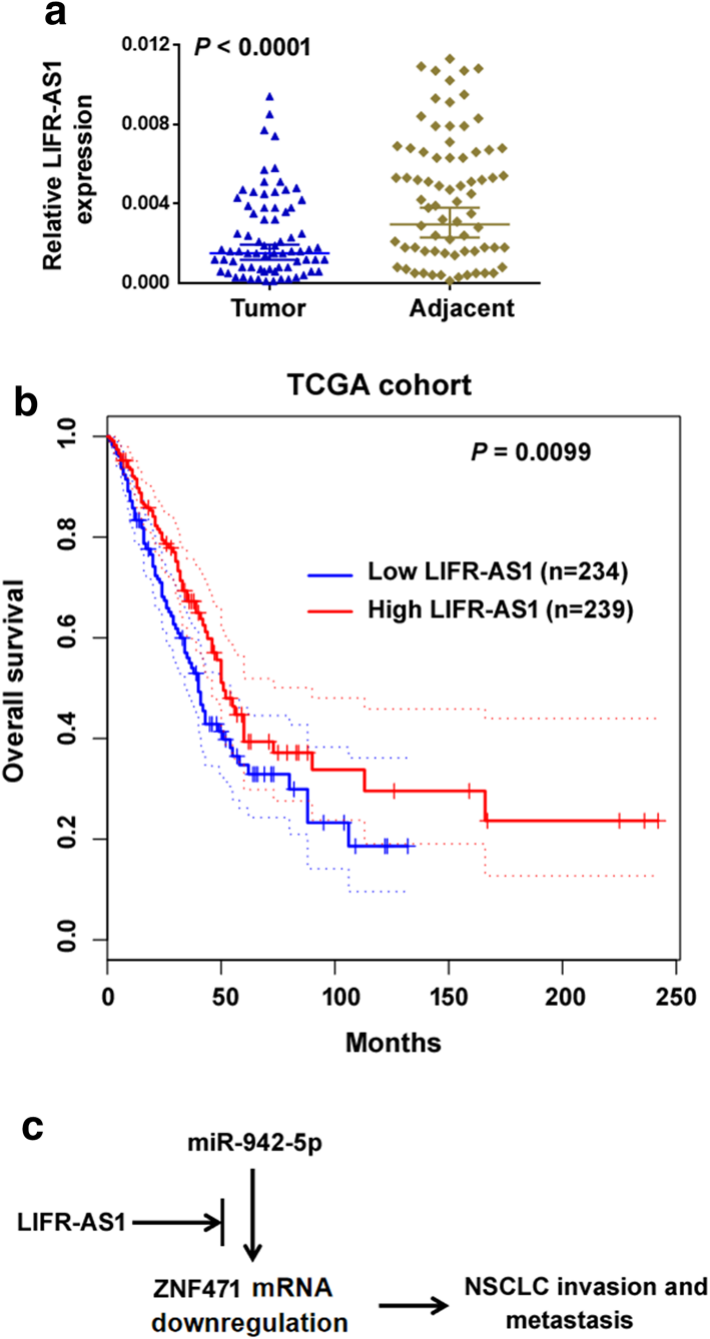
Clinical significance of LIFR-AS1 in NSCLC. **a** Measurement of the expression levels of LIFR-AS1 in 73 pairs of NSCLC and corresponding noncancerous tissues. **b** Kaplan–Meier analysis based on lung adenocarcinoma TCGA dataset revealed that low expression of LIFR-AS1 was significantly correlated with a reduced overall survival. Statistical differences were determined by the log-rank test. **c** Schematic model showing that LIFR-AS1 sponges miR-942-5p to derepress ZNF471, consequently blocking NSCLC cell invasion and metastasis

To investigate the clinical relevance of LIFR-AS1 expression in NSCLC, we measured the expression levels of LIFR-AS1 in 73 pairs of NSCLC and corresponding noncancerous tissues. We observed that LIFR-AS1 expression was downregulated in the NSCLC tissues compared with adjacent normal tissues (*P* < 0.0001; Fig. 6a). The downregulation of LIFR-AS1 was significantly correlated with advanced TNM stage (*P* = 0.0321) and lymph node metastasis (*P* = 0.0047; Table 1). Kaplan–Meier analysis based on lung adenocarcinoma TCGA dataset revealed that low expression of LIFR-AS1 was significantly correlated with a reduced overall survival (*P* = 0.0099; Fig. 6b). These data demonstrate that LIFR-AS1 expression is a favorable prognostic factor in NSCLC.

## Discussion

In this work, we validate LIFR-AS1 as a key miR-942-5p-interacting lncRNA in NSCLC cells. LIFR-AS1 carries the binding site for miR-942-5p, and overexpression of miR-942-5p decreases the expression of LIFR-AS1 in NSCLC cells. Analysis of TCGA data reveals a negative correlation between miR-942-5p and LIFR-AS1 in lung adenocarcinoma (Additional file 1: Figure S5). Given that miR-942-5p has tumor-promoting effects on NSCLC [17], we speculated that LIFR-AS1 may play an important role in NSCLC progression. In line with this hypothesis, we show that LIFR-AS1 is downregulated in NSCLC tissues and cells, and ectopic expression of LIFR-AS1 leads to an inhibition of NSCLC cell migration and invasion. Conversely, depletion of LIFR-AS1 enhances the migration and invasion of NSCLC cells. These results point toward LIFR-AS1 as a tumor suppressor in NSCLC.

Dysregulation of lncRNAs is frequently detected in cancers, suggesting the potential as a biomarker for cancer progression [7–9]. Our data indicate that downregulation of LIFR-AS1 is significantly associated with advanced TNM stage and lymph node metastasis of NSCLC patients. Moreover, LIFR-AS1 downregulation predicts poor prognosis in NSCLC. Therefore, LIFR-AS1 may serve as a novel prognostic marker for NSCLC. The clinical findings also suggest the therapeutic potential of LIFR-AS1 in preventing NSCLC progression.

It has been suggested that lncRNAs can function as ceRNA for miRNAs to modulate diverse biological processes [26, 27]. For example, Xu et al. [27] reported that the lncRNA SPRY4-IT1 promotes cell proliferation, migration, and invasion in cholangiocarcinoma by sponging miR-101-3p. Having identification of the interaction between LIFR-AS1 and miR-942-5p, we checked whether LIFR-AS1 exerts its tumor-suppressive effects through inhibition of miR-942-5p activity. Interestingly, we find that LIFR-AS1-mediated inhibition of NSCLC cell migration and invasion is rescued by ectopic expression of miR-942-5p. Biochemically, both LIFR-AS1 and miR-942-5p are enriched in Ago2 immunoprecipitates from NSCLC cells. These results suggest that sponging miR-942-5p accounts for the anti-invasive activity of LIFR-AS1 in NSCLC.

It has been documented that miR-942-5p can target BARX2 in NSCLC [17]. However, we did not detect the regulation of BARX2 by LIFR-AS1. This result suggest that LIFR-AS1 likely interferes with miR-942-5p binding to other target mRNAs. This hypothesis is supported by the finding that LIFR-AS1 can prevent the repression of ZNF471 by miR-942-5p. We provide first evidence that ZNF471 is a direct target gene of miR-942-5p. RIP assays reveal that miR-942-5p overexpression leads to an enrichment of ZNF471 mRNA in Ago2 immunoprecipitates. Enforced expression of miR-942-5p significantly decreases the expression of ZNF471 in NSCLC cells, whereas overexpression of LIFR-AS1 causes an upregulation of ZNF471. In addition, LIFR-AS1 is positively correlated with ZNF471 mRNA in lung adenocarcinoma, according to TCGA datasets (Additional file 1: Figure S6). It has been reported that ZNF471 can block the proliferation and invasion of gastric cancer cells [5]. Consistently, we also validate ZNF471 as a tumor suppressor in NSCLC. We show that overexpression of ZNF471 restrains the invasion of PC-9 cells. Moreover, depletion of ZNF471 or overexpression of miR-942-5p restores the invasive property in LIFR-AS1-overexpressing NSCLC cells. Taken together, we propose a model in which LIFR-AS1 sponges miR-942-5p to derepress ZNF471, consequently blocking NSCLC cell invasion and metastasis (Fig. 6c).

## Conclusion

Our findings indicate that LIFR-AS1 acts as a sponge of miR-942-5p and prevents miR-942-5p-mediated repression of ZNF471, consequently impairing NSCLC cell invasion and metastasis. LIFR-AS1 downregulation is associated with poor prognosis of NSCLC patients. Therefore, we suggest LIFR-AS1 as a possible therapeutic target for NSCLC.

## Supplementary information

**Additional file 1: Table S1.** Prediction of miR-942-5p-interacting lncRNAs with using the Encyclopedia of RNA Interactomes (ENCORI) program. **Figure S1.** Effect of LIFR-AS1 overexpression on the expression of miR-942-5p in NSCLC cells. n.s. indicates no significance. **Figure S2.** Effect of LIFR-AS1 overexpression on the expression of BARX2 in NSCLC cells. (A) Measurement of BARX2 mRNA levels. (B) Western blot analysis of BARX2 protein levels. n.s. indicates no significance. **Figure S3.** Effect of miR-942-5p overexpression on the abundance of RND3, KDM5A, and CCBE1 mRNAs in NSCLC cells. n.s. indicates no significance. **Figure S4.** Effect of LIFR-AS1 overexpression or knockdown on the proliferation of (A) PC-9 and (B) A549 cells. Cell proliferation was determined by direct cell counting at indicated time points after plating. n.s. indicates no significance. **Figure S5.** Analysis of TCGA data reveals a negative correlation between miR-942-5p and LIFR-AS1 in lung adenocarcinoma. **Figure S6.** Analysis of TCGA data reveals a positive correlation between LIFR-AS1 and ZNF471 mRNA in lung adenocarcinoma.

## Data Availability

Not applicable

## Abbreviations

ceRNA: Competitive endogenous RNA
lncRNA: Long non-coding RNA
NSCLC: Non-small cell lung cancer
qRT-PCR: Quantitative real-time PCR
shRNA: Short hairpin RNA
UTR: Untranslated region
ZNF471: Zinc-finger protein 471
