# Supplementary material for: lncRNA LIFR-AS1 suppresses invasion and metastasis of non-small cell lung cancer via the miR-942-5p/ZNF471 axis
Source: Cancer Cell Int. 2020 May 24;20:180. doi: 10.1186/s12935-020-01228-5 (PMC7245777; doi:10.1186/s12935-020-01228-5)
Supplement: Supplementary file 1 — Additional file 1: Table S1. Prediction of miR-942-5p-interacting lncRNAs with using the Encyclopedia of RNA Interactomes (ENCORI) program. Figure S1. Effect of LIFR-AS1 overexpression on the expression of miR-942-5p in NSCLC cells. n.s. indicates no significance. Figure S2. Effect of LIFR-AS1 overexpression on the expression of BARX2 in NSCLC cells. (A) Measurement of BARX2 mRNA levels. (B) Western blot analysis of BARX2 protein levels. n.s. indicates no significance. Figure S3. Effect of miR-942-5p overexpression on the abundance of RND3, KDM5A, and CCBE1 mRNAs in NSCLC cells. n.s. indicates no significance. Figure S4. Effect of LIFR-AS1 overexpression or knockdown on the proliferation of (A) PC-9 and (B) A549 cells. Cell proliferation was determined by direct cell counting at indicated time points after plating. n.s. indicates no significance. Figure S5. Analysis of TCGA data reveals a negative correlation between miR-942-5p and LIFR-AS1 in lung adenocarcinoma. Figure S6. Analysis of TCGA data reveals a positive correlation between LIFR-AS1 and ZNF471 mRNA in lung adenocarcinoma. [file 12935_2020_1228_MOESM1_ESM.docx]

**Additional data**

Table S1. Prediction of miR-942-5p-interacting lncRNAs with using the Encyclopedia of RNA Interactomes (ENCORI) program

| **Gene** | **Genbank number** |
| --- | --- |
| MALAT1 | NR_002819.4 |
| NORAD | NR_027451.1 |
| XIST | NR_001564.2 |
| NEAT1 | EF177379.1 |
| MIR497HG | NR_038310.1 |
| LINC00943 | NR_038256.1 |
| SNHG15 | NR_003697.2 |
| NUTM2A-AS1 | NR_024397.1 |
| HCG11 | NR_026790.1 |
| ZNF674-AS1 | NR_015378.3 |
| PKP4-AS1 | NR_135242.1 |
| LINC01176 | NR_108081.1 |
| HCG18 | NR_024052.2 |
| LINC01087 | NR_108087.1 |
| NIFK-AS1 | NR_037856.1 |
| NNT-AS1 | NR_073113.1 |
| LIFR-AS1 | NR_103553.1 |
| CTBP1-AS2 | NR_033339.1 |
| THUMPD3-AS1 | NR_132780.1 |
| LINC00963 | NR_038955.1 |
| MIR100HG | NR_024430.2 |


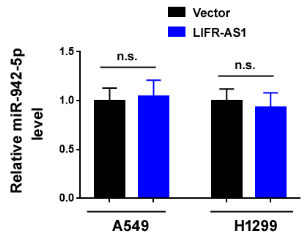


Figure S1. Effect of LIFR-AS1 overexpression on the expression of miR-942-5p in NSCLC cells. n.s. indicates no significance.


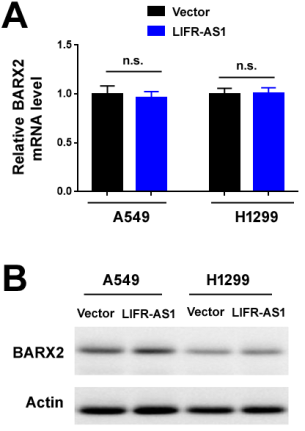


Figure S2. Effect of LIFR-AS1 overexpression on the expression of BARX2 in NSCLC cells. (A) Measurement of BARX2 mRNA levels. (B) Western blot analysis of BARX2 protein levels. n.s. indicates no significance.


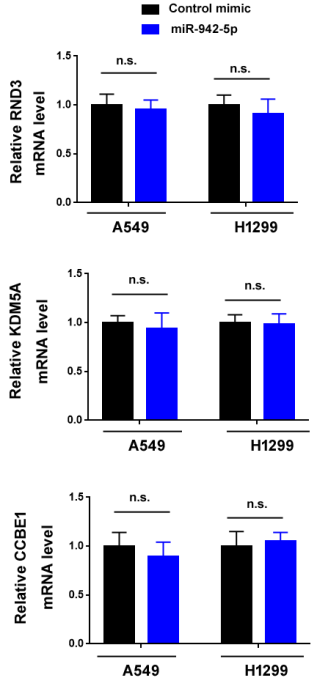


Figure S3. Effect of miR-942-5p overexpression on the abundance of RND3, KDM5A, and CCBE1 mRNAs in NSCLC cells. n.s. indicates no significance.


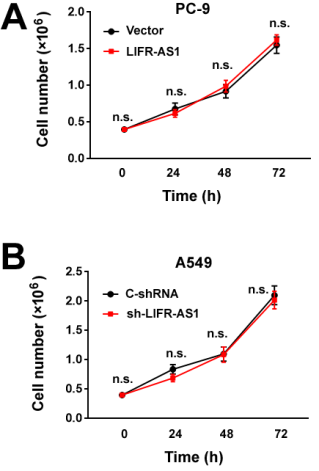


Figure S4. Effect of LIFR-AS1 overexpression or knockdown on the proliferation of (A) PC-9 and (B) A549 cells. Cell proliferation was determined by direct cell counting at indicated time points after plating. n.s. indicates no significance.


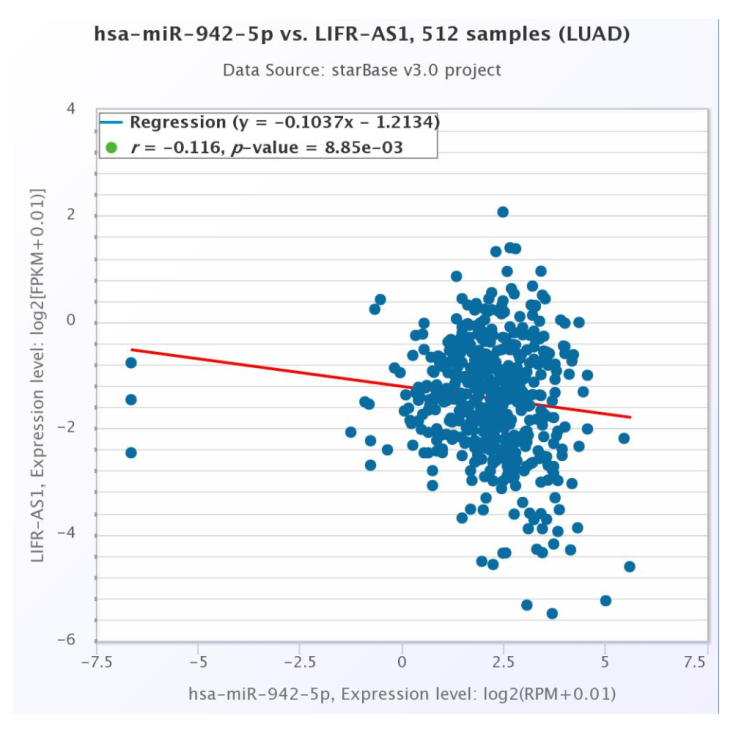


Figure S5. Analysis of TCGA data reveals a negative correlation between miR-942-5p and LIFR-AS1 in lung adenocarcinoma.


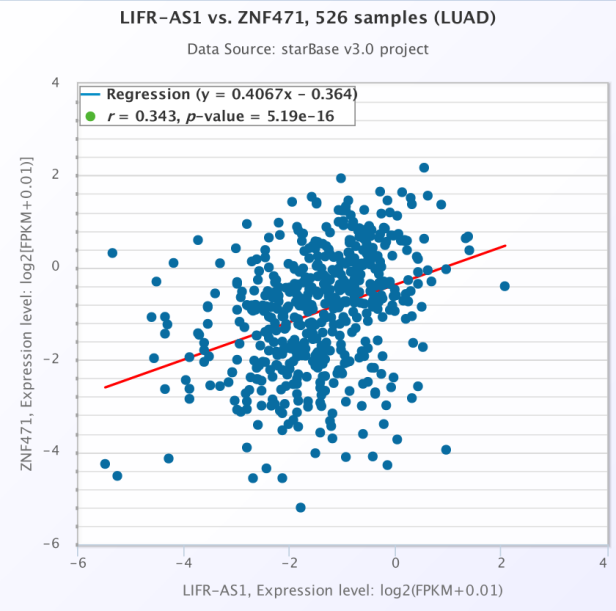


Figure S6. Analysis of TCGA data reveals a positive correlation between LIFR-AS1 and ZNF471 mRNA in lung adenocarcinoma.
